# Supplementary material for: Serum Complement C4 Levels Are a Useful Biomarker for Predicting End-Stage Renal Disease in Microscopic Polyangiitis
Source: Int J Mol Sci. 2023 Sep 22;24(19):14436. doi: 10.3390/ijms241914436 (PMC10572948; doi:10.3390/ijms241914436)
Supplement: Supplementary file 1 [file ijms-24-14436-s001.zip › ijms-2609605-supplementary.pdf]

Supplementary Table S1. The contexts of systemic symptoms and initial treatment in patients with MPA

Characteristics	MPA (n= 74)
Systemic Symptoms	
General, n (%)	47 (63.5)
Cutaneous, n (%)	12 (16.2)
Mucous membrane, n (%)	8 (10.8)
Ear, nose, throat, n (%)	15 (20.3)
Chest, n (%)	24 (32.4)
Cardiovascular, n (%)	5（6.8）
Abdominal, n (%)	1 (1.4)
Renal, n (%)	57 (77.0)
Nervous system, n (%)	38 (51.4)
Initial treatment	
PDN, mg/day	50 (40-60)
MPDN pulse, n (%)	20 (27.0)
Immunosuppressants	
IVCY, n (%)	41 (55.4)
Total IVCY dose (g)	1.4 (0.6-2)
RTX, n (%)	10 (13.5)
IVIG, n (%)	2 (2.7)
AZA/MTX/ TAC/MZB, n (%)	50 (67.6)/2(2.7)/ 4(5.4)/ 1 (1.4)

The laboratory markers are presented as the median (interquartile range). MPA: microscopic polyangiitis; PDN: prednisolone; MPDN: methylprednisolone; IVCY: intravenous cyclophosphamide; RTX: rituximab; IVIG: intravenous immunoglobulin; AZA: azathioprine; MTX: methotrexate; TAC: tacrolimus; MZB: mizoribine.
